# Reassessing Drought Tolerance in Citrus Tetraploid Rootstocks: Myth or Reality?

**DOI:** 10.1111/ppl.70199

**Published:** 2025-04-02

**Authors:** Lucas da Silva Costa, Luciano Freschi, Maurício Antonio Coelho Filho, Monique Ayala Araújo da Silva, Fernanda dos Santos Nascimento, Abelmon da Silva Gesteira

**Affiliations:** ^1^ Departamento de Biologia, Centro de Genética e Biologia Molecular Universidade Estadual de Santa Cruz Ilhéus Bahia Brasil; ^2^ Departamento de Botânica Instituto de Biociências, Universidade de São Paulo Brasil; ^3^ Embrapa Mandioca e Fruticultura, Cruz das Almas, Bahia Brasil

## Abstract

Polyploidy, particularly tetraploidy, has emerged as a promising tool in citrus rootstock breeding due to its potential to enhance drought tolerance. This review examines the role of tetraploid rootstocks in drought resilience, focusing on molecular and physiological adaptations observed in controlled environments and field conditions. Tetraploids display traits such as increased abscisic acid (ABA) production, antioxidant defenses, and osmotic adjustments. However, these advantages often fail to translate into superior drought tolerance in field conditions, where competition for resources and environmental complexities significantly influence plant responses. Recent evidence suggests that methodological limitations in earlier studies, particularly in pots, may have overstated the benefits of tetraploids. Field studies indicate that diploids, with more extensive root systems and greater water extraction capacity, often outperform tetraploids under water stress. To advance citrus breeding, it is essential to standardize experimental approaches, control soil matric potential, and prioritize long‐term studies. Identifying key genes and metabolic pathways associated with drought tolerance, along with the application of advanced tools such as CRISPR/Cas9, will enable the development of resilient rootstocks, ensuring sustainable citrus production amidst increasing water scarcity and climate change.

## 1 INTRODUCTION

The *Citrus* genus, of great economic and nutritional importance, has been the focus of various research aiming to improve its characteristics (Abobatta, 2023; Zhong and Nicolosi, 2020). In this context, polyploidy, a phenomenon that involves the acquisition of more than two complete sets of chromosomes, emerges as a promising tool for the genetic improvement of citrus. Polyploidy, recurrent in plant evolution, despite initially causing instability, provides raw material for evolution, allowing the diversification and specialization of genes (Leitch and Leitch, 2008; Ramsey, 2011; Soltis et al. 2015; Trojak‐Goluch et al. 2021). In particular, tetraploid plants, characterized by four sets of chromosomes in each cell, have attracted significant interest in citrus farming. Studies suggest that these plants may exhibit greater robustness and resilience to a variety of stresses, including drought (Aleza et al. 2009; Jiang et al. 2022; Jokari et al. 2022; Oustric et al. 2019; Ruiz et al. 2018; Sivager et al. 2021; Wu et al. 2005).

Drought is one of the main challenges citrus farming faces, which has intensified due to climate change and increasing water scarcity (Arora, 2019; Dietz et al. 2021). The search for tolerant varieties has thus become a crucial priority for the sustainability of citrus production. Rootstocks, essential components in citrus farming, play a vital role by influencing the growth, productivity, and adaptation of citrus plants to different environmental conditions, including drought (Balfagón et al. 2022; Santana‐Vieira et al. 2016; Santos et al. 2019). In this context, the search for drought‐tolerant rootstocks emerges as a fundamental pillar to ensure the productivity and sustainability of the crop, especially in regions with limited or unpredictable water resources.

Recent studies have suggested that tetraploid rootstocks may represent a promising alternative for improving drought tolerance in citrus. This potential tolerance has been associated with various adaptive responses, such as changes in the expression of genes related to osmotic regulation, protection against oxidative damage, and maintenance of photosynthesis under water deficit, as well as the activation of antioxidant enzymes and osmotic adjustment (Allario et al. 2013; Chen, 2007; Ollitrault et al. 2020). However, the assumption that tetraploids unequivocally outperform diploids in drought tolerance requires careful evaluation. Evaluations should consider the complex genotype‐environment interactions, particularly in pot experiments where the limited water volume can result in diploid rootstocks depleting water faster than tetraploids due to higher transpiration rates (Khalid et al. 2021; Syvertsen et al. 2000). These interactions, when not taken into account, can lead to misinterpretations, as variations in soil moisture depletion and stress levels are influenced by the restricted water availability in pots (Ogbaga et al. 2020).

The understanding of the molecular and physiological mechanisms involved in the drought tolerance of tetraploid rootstocks is still limited, especially under field conditions, where plants are subject to a wider variety of environmental factors, such as temperature, solar radiation, and, crucially, the soil matric potential, which influences water availability to the roots (Atkinson and Urwin, 2012). Comparatively, controlled environments like growth chambers or greenhouses do not faithfully reproduce this dynamic. It is essential to consider the matric potential in drought tolerance studies, especially in pots, to approximate experimental conditions to field reality (da Silva Costa et al. 2024; Ogbaga et al. 2020; Sousa et al. 2024). Furthermore, understanding the interaction between tetraploid rootstocks and scion varieties under water deficit stress, alongside comparisons with diploids, is vital for developing effective management strategies. These insights will support the selection of rootstock‐scion combinations that maximize productivity and sustainability in citrus cultivation (Abobatta, 2023; da Silva Costa et al. 2024; Jiang et al. 2022).

This review aims to critically analyze the existing evidence on the impact of tetraploidization on drought tolerance in citrus rootstocks, with a particular focus on comparing studies conducted in pots and under field conditions. Additionally, the physiological and biochemical mechanisms underlying drought tolerance, the relevance of soil matric potential in this assessment, and the practical implications for citrus cultivation will be discussed. By integrating these diverse perspectives, the goal is to contribute to the advancement of knowledge on tetraploidization in citrus and provide crucial information for the development of more drought‐resilient rootstocks, a vital challenge for the sustainability of citrus cultivation in the face of increasing water scarcity.

### 1.1 The Tetraploidization Process and Its Impact on Plant Development

Plant polyploidy is a phenomenon of great evolutionary and agronomic importance that has been studied for nearly a century. The first works on polyploidy began with the pioneering research of Hugo de Vries in the early 20th century, when he discovered tetraploidy in various plant species (Lutz, 1907; De Vries, 2023). This initial discovery led to a series of investigations that revealed the prevalence and diversity of polyploidy in the plant kingdom, establishing the foundations for the current understanding of its mechanisms, consequences, and applications (Jackson, 1976; Sattler et al. 2016; Stebbins, 1950).

Polyploidy, characterized by more than two complete sets of chromosomes in an organism, plays a crucial role in genetic diversification and the evolution of many plant species. Among them, citrus is a notable example, with research demonstrating the importance of polyploidy for its diversity and adaptation (Carputo and Aversano, 2016; Guerra et al. 2016; Khan, 2007; Lee, 1988; Ollitrault et al. 2007; Wang et al. 2018).

**FIGURE 1:**
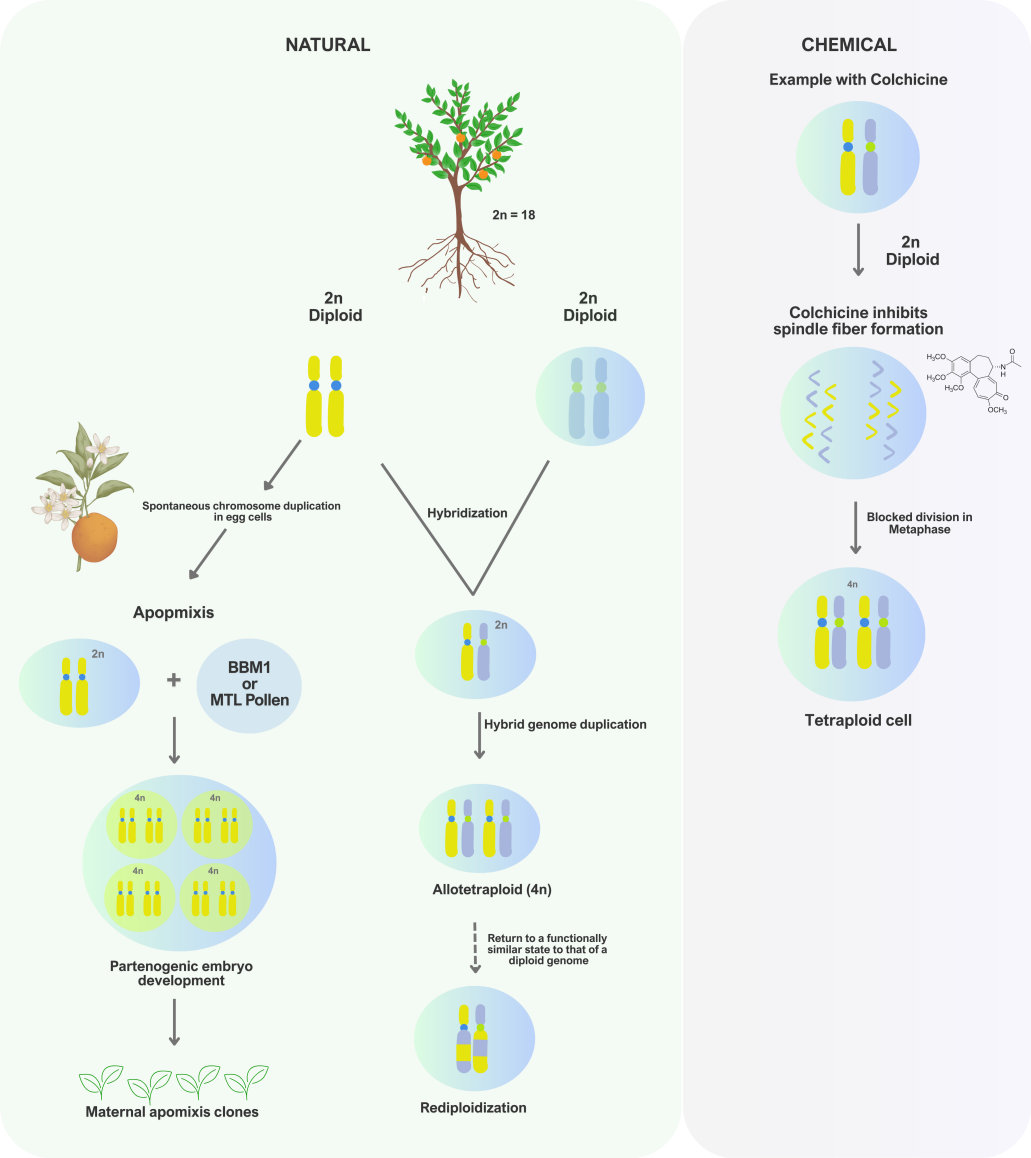
Cytological mechanisms of polyploidization in plants, including both natural and chemical processes. In citrus, apomixis predominantly occurs as adventitious embryony, where a single seed can contain both a zygotic embryo and multiple apomictic embryos of nucellar origin. These apomictic embryos give rise to maternal clones, which are typically diploid (2n) and genetically identical to the mother plant. However, spontaneous chromosome duplication in diploid egg cells (2n) can produce tetraploid embryos (4n), involving genes such as BBM1 and MTL, which promote embryo development without fertilization. During hybridization, the union of diploid gametes (2n) can generate a hybrid genome that, after chromosome duplication, forms allotetraploids (4n). These allotetraploids may undergo rediploidization, functionally returning to a diploid state. In the chemical process, colchicine inhibits spindle fiber formation during metaphase, blocking chromosome segregation and resulting in tetraploid cells (4n).

Polyploidy can be classified into two main types: autopolyploidy and allopolyploidy (Figure 1), which result from somatic chromosomal duplication or sexual reproduction via 2n gametes, respectively (Allario et al. 2013; Stebbins, 1947). Autopolyploidy occurs through the duplication of the genome of a single ancestral genotype, resulting in individuals with multiple copies of each chromosome. Although autopolyploids often show little morphological differentiation from their diploid progenitors, meiosis can be problematic due to the formation of multivalents, which can cause incorrect chromosomal segregation and negatively affect fertility (Bomblies et al. 2016; Madlung, 2013; Ramsey and Schemske, 2002). However, individuals with an even number of chromosome sets, such as autotetraploids, tend to exhibit greater meiotic stability compared to polyploids with an odd number (3n, 5n, etc.), as homologous chromosomes more regularly form bivalent pairs, facilitating balanced segregation of chromosomes to the daughter cells (Gaeta and Pires, 2010).

In contrast, allopolyploidy results from hybridization between different species or genotypes followed by duplication of the hybrid genome (Mason and Wendel, 2020). Chromosomal duplication restores fertility and allows regular chromosomal pairing during meiosis (Chen, 2010). Allopolyploids often exhibit novel morphological and physiological characteristics, combining traits from both parental genotypes (Soltis and Soltis, 1999). This genetic diversification, driven by the duplication of parental genomes, can lead to new traits and adaptations in dynamic and ever‐changing environments (Leitch and Leitch, 2008; Parisod et al. 2010).

After a whole genome duplication (WGD) event, such as tetraploidization, plants often enter a process of rediploidization (Figure 1). This process is not simply a return to a functional diploid state but a complex and crucial step that allows the plant to reorganize and stabilize its genome while maintaining the additional genetic richness acquired during tetraploidization. The presence of tetraploids can thus be interpreted as an indication that the plant is in an intermediate stage of evolutionary adaptation, where the process of rediploidization is ongoing. This process is not just an attempt to stabilize the genome but also to optimize fertility, ensuring that newly acquired adaptive traits are effectively transmitted to future generations (Chen, 2015; Sankoff and Zheng, 2018).

Rediploidization involves the loss of duplicated genes, chromosomal rearrangements such as fusions and fissions, and descending diploidization, which reduces the number of chromosomes through translocations (Mandáková and Lysak, 2018). This process stabilizes the genome and facilitates the retention of genes that confer adaptive advantages, promoting genetic diversity. Polyploidy provides raw material for evolution, allowing the emergence of new genes through mutations and rearrangements, while genetic redundancy in tetraploids can increase tolerance to deleterious mutations, enabling the exploration of new ecological niches (Magadum et al. 2013; Otto and Yong, 2002). Evolutionarily, tetraploidy can offer immediate advantages, such as greater vigor or stress tolerance, which is crucial in challenging environments. The true importance of rediploidization, however, lies in its ability to promote genomic flexibility, allowing the plant to explore new metabolic pathways, gene functions, and morphological characteristics.

Currently, many citrus genotypes are analyzed as if the differences resulting from tetraploidy, compared to diploid individuals, were merely adaptive variations (Allario et al. 2013; Oliveira et al. 2017; Oustric et al. 2019; Ruiz et al. 2016). However, this perspective is often influenced by experimental limitations that may have led to misinterpretations, underestimating the crucial role of rediploidization. These misconceptions have led to an overestimation of the adaptability of tetraploid individuals relative to diploids, without adequately considering the genomic flexibility promoted by rediploidization, which plays an essential role in the evolution and adaptation of species.

Polyploidy can occur naturally through the failure of chromosome separation during meiosis, resulting in unreduced gametes or through the fusion of unreduced gametes (Leitch and Leitch, 2008). Additionally, tetraploidy can be artificially induced through somatic cell fusion or the use of chemical agents, such as oryzalin, trifluralin, and colchicine (Yemets and Blume, 2008). Colchicine (C₂₂H₂₅NO₆), a widely used antimitotic agent in plant breeding, is the most effective substance for inducing chromosomal duplication (Eeckhaut et al. 2004; Eng and Ho, 2019; Revathi and Thomas, 2022). It directly affects the formation of the mitotic spindle during cell division, preventing proper chromosome separation and resulting in cells with double the usual number of chromosomes (Figure 1), characterizing tetraploidy (Eng et al. 2021; Yan et al. 2022). Recent research has explored the use of colchicine to induce tetraploidy in Citrus genotypes in a laboratory environment (*in vitro*) (Cimen, 2020; Narukulla et al. 2023; Yasuda et al. 2022), aiming to enhance the characteristics and productive potential of the plants.

Apomixis, a common asexual reproductive process in citrus (Wang et al. 2022), also plays an important role in the generation of polyploids, especially tetraploids, through the spontaneous duplication of chromosomes in nucellar cells of the ovule (Figure 1). Studies by Aleza et al. (2011) and Dang et al. (2024) confirm the viability of obtaining tetraploids in citrus by combining apomixis with spontaneous chromosomal duplication. This strategy offers a promising alternative for developing new varieties with improved characteristics, taking advantage of the benefits of polyploidy and apomixis.

Tetraploidy, characterized by the duplication of the number of chromosomes and the amount of DNA, leads to an increase in cell size, including the volume of cytoplasm and organelles (Aleza et al. 2009; Bhuvaneswari et al. 2020; Tan et al. 2015). Additionally, tetraploidy affects the phenotypic and morphological characteristics of the plant. These phenotypic modifications can influence both productivity and tolerance to environmental stresses, which can provide adaptive advantages under certain conditions (Tan et al. 2015).

Tetraploidy generally induces a reduction in plant canopy size, resulting in a dwarf or semi‐dwarf phenotype. As demonstrated by Azevedo et al. (2015, 2020), this characteristic can be highly advantageous in terms of cultivation, especially in situations of limited space or when optimizing management and harvesting is desired. Plants with smaller canopies allow for denser planting, increasing productivity per area. Furthermore, the reduction in canopy size facilitates the application of agricultural pesticides, fruit harvesting, and pruning, contributing to reduced production costs.

In addition to the reduction in canopy size, tetraploidy can induce other significant morphological changes, such as the development of larger flowers with more intense colors, larger fruits with higher nutrient content, and more robust stems and roots (Hu et al. 2021; Jaskani et al. 2002; Wu et al. 2012). These changes are attributed to the dosage effect, where the increase in the number of chromosome sets (from diploid to tetraploid) results in a greater amount of genetic material, directly influencing the phenotypic characteristics of the plants (Doyle and Coate, 2019).

### 1.2 Rootstock‐mediated mechanisms modulating citrus drought tolerance

The drought‐induced effects on citrus plant development vary among different scion/rootstock combinations (Carr, 2012; Romero et al. 2006; Santana‐Vieira et al. 2016; Shafqat et al. 2021). During drought, plants must balance photosynthesis and water conservation. Stomatal closure helps prevent water loss by reducing transpiration but also limits CO2 entry, which is essential for photosynthesis. Studies show an inverse relationship between stomatal conductance and drought tolerance: closure increases water retention, while opening increases transpiration and reduces tolerance (García‐Sánchez et al. 2007; Manacorda et al. 2021). The timing and intensity of stomatal closure are critical for survival, with different strategies balancing water economy and photosynthesis (Ilyas et al. 2021; Martin‐StPaul et al. 2017; Rodriguez‐Dominguez et al. 2016). Imbalances in water uptake and transpiration cause turgor loss, leading to wilting symptoms, such as reduced leaf and stem rigidity, indicating water deficit (Shafqat et al. 2021; Yang et al. 2021).

Among the hormonal cues, abscisic acid (ABA) plays a central role in regulating stomatal closure. Under drought conditions, ABA concentration increases in plant roots and leaves. Upon water deficit, ABA is synthesized in the roots through the action of key genes in the ABA biosynthetic pathway, including 9‐cis‐epoxycarotenoid dioxygenase 2 (*NCED2*), *NCED3*, and abscisic aldehyde oxidase 3 (*AAO3*). These genes are activated by the transcriptional regulator MYB‐like protein MAD23, which enhances the synthesis of protective compounds (Ali et al. 2020; Manzi et al. 2015; Manzi et al. 2016; Pedrosa et al. 2017; Zhang et al. 2023). The synthesized ABA is then transported to the leaves through the xylem, where it regulates stomatal opening by acting on guard cells (Ali et al. 2020; Muhammad Aslam et al. 2022). ABA acts as a long‐distance chemical signal from roots to shoots, increasing in concentration during drought stress, which leads to stomatal closure to reduce water loss (Forner‐Giner et al. 2011).

In the leaves, either ABA synthesized locally or transported from the roots interact with receptors from the pyrabactin resistance/pyrabactin‐like/receptor for activated C kinase (PYR/PYL/RCAR) family, such as CsPYL4 and CsPYL5. This interaction initiates a signaling cascade that includes the activation of phosphatases like type 2C protein phosphatase CsPP2CA, abscisic acid insensitive 1 (*ABI1*), and *ABI2*, which facilitate the transduction of ABA stress signals (Gonçalves et al. 2019; Jiang et al. 2014; Romero et al. 2012). These signals lead to the activation of various transcription factors, including WRKY40, NAC domain‐containing protein 2 (NAC2), and AP2/ERF transcription factors, which regulate genes involved in redox adjustments and protection against reactive oxygen species (ROS) that accumulate under stress (Gonçalves et al., 2019; Romero et al. 2012; Santos et al. 2021).

Studies on citrus have shown that sensitivity to ABA signaling varies among rootstocks, influencing drought adaptation efficiency (Santana‐Vieira et al. 2016; Zandalinas et al. 2016). Some varieties may be more sensitive to ABA, closing their stomata more quickly in response to drought stress. Santos et al. (2020) demonstrated that the Tahiti acid lime (TAL) variety exhibits pronounced insensitivity to ABA, with the majority of its stomata remaining open (61.4% to 67.6%) even under high concentrations of exogenous ABA. This behavior contrasts with varieties like Valencia Orange (VO), which show significant stomatal closure under similar conditions. These findings, supported by both quantitative and photographic evidence, underscore the importance of exploring ABA sensitivity as a factor in drought tolerance.

**FIGURE 2:**
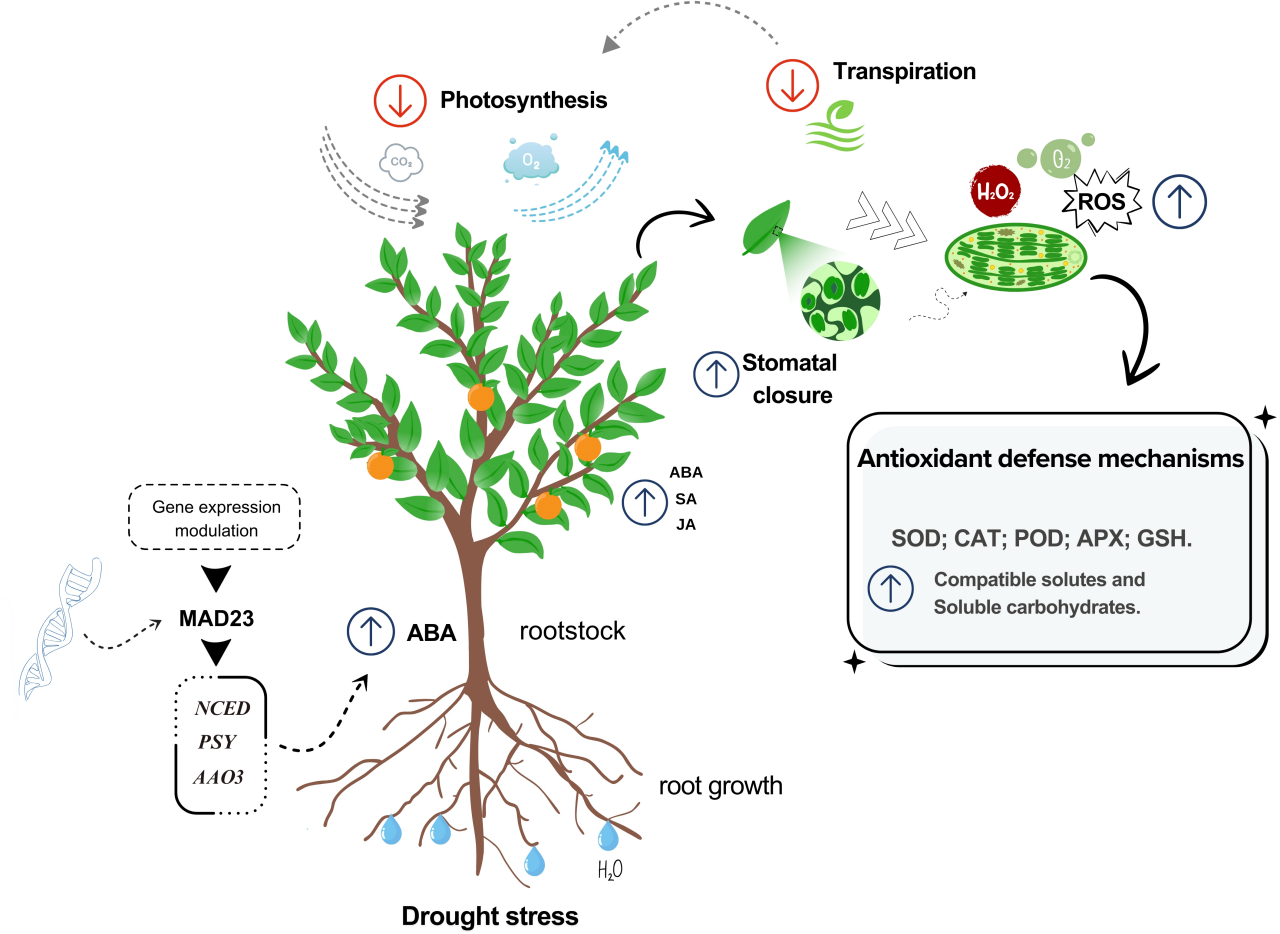
Citrus plant response to water stress: Under drought conditions, citrus plants activate adaptive mechanisms to ensure survival. The transcriptional regulator MAD23 triggers the expression of specific genes (NCED, PSY, and AAO3), increasing the production of abscisic acid (ABA), a hormone that induces stomatal closure, thus reducing transpiration and conserving water, albeit at the cost of lower photosynthetic rates. Simultaneously, water stress elevates the levels of salicylic acid (SA) and jasmonic acid (JA), which also play crucial roles in modulating the stress response. Water stress also increases the production of reactive oxygen species (ROS), which can be harmful at high concentrations. To neutralize these effects, the plant enhances the activity of antioxidant enzymes such as SOD, CAT, POD, APX, and GSH, and synthesizes compatible solutes and soluble carbohydrates to maintain osmotic balance and protect cells. The response also includes root growth directed towards deeper soil layers to access water, promoting plant resilience.

In addition to regulating stomatal closure, ABA also stimulates the expression of genes encoding heat shock proteins (HSPs) and antioxidant enzymes, including superoxide dismutase, catalase, and peroxidase (Figure 2). These enzymes neutralize excess reactive oxygen species (ROS) produced under drought stress (Neves et al. 2017; Santos et al. 2019; Santos et al. 2021; Xian et al. 2014; Yang et al. 2024). Antioxidant capacity is crucial for protecting cells from oxidative damage caused by ROS, which can lead to lipid peroxidation, protein and DNA damage, culminating in cell death (Hussain et al. 2019).

Gonçalves et al. (2019) investigated the molecular responses induced by rootstocks associated with drought tolerance in sweet oranges. They observed that drought tolerance induced by the rootstock in sweet oranges involves the transcriptional activation of genes related to cell wall metabolism, soluble carbohydrates, and antioxidants. The study also revealed a positive drought‐triggered regulation of genes encoding HSPs, ABA signaling components, and ABA‐responsive members of the DEMETER‐like DNA demethylases (DMLs) family. DMLs participate in active DNA demethylation, an epigenetic process that can influence gene expression and stress response. ABA also plays a crucial role in regulating citrate accumulation in citrus fruits, affecting their acidity. Under drought, the expression of genes encoding a bHLH transcription factor (*CsAN1*) and a P3A‐ATPase (*CsPH8*), important in regulating citrate accumulation, is significantly increased through the activating the ABA‐responsive element binding factor 3 (*CsABF3*) (Ma et al. 2024).

In addition to ABA, other hormones such as jasmonic acid (JA) and salicylic acid (SA) also play crucial roles in mediating plant responses to drought (De Ollas et al. 2013; Gupta et al. 2021; Santana‐Vieira et al., 2016). Both JA and SA enhance the antioxidative capacity of plant cells, reducing lipid peroxidation and maintaining membrane integrity under drought conditions. This is achieved by increasing the activity of antioxidant enzymes and the accumulation of osmolytes like proline and soluble carbohydrates, which help in stress mitigation (Ghassemi‐Golezani and Farhangi‐Abriz, 2021; Ababaf et al. 2021).

Furthermore, JA and SA interact with ABA in a complex hormonal crosstalk. JA transiently accumulates in response to drought, which is necessary for the subsequent increase in ABA levels, suggesting a regulatory role of JA in ABA biosynthesis under stress conditions. However, under prolonged drought, reductions in JA levels have been observed. For example, in drought‐stressed *Citrus wilsonii*, JA and JA‐Ile levels significantly decreased to approximately 6% and 4% of their control levels in diploids, and to around 12% and 3% in tetraploids, respectively (Jiang et al. 2022). This suggests that the role of JA may shift as drought stress persists, moving from an initial accumulation to a marked reduction in more prolonged conditions.

Salicylic acid (SA) helps plants cope with drought stress by balancing the hormonal pathways of ABA and JA, reducing their levels and restoring hormonal harmony. JA rapidly increases in response to water stress, promoting ABA accumulation and stomatal closure (De Ollas et al., 2013). Additionally, JA affects stomatal conductance and volatile emissions, highlighting its role in stress signaling (Jiang et al., 2021). On the other hand, SA suppresses JA signaling by altering the expression of JA‐related genes and potentially modifying stomatal responses (Does et al., 2013). Under drought stress, SA levels increase significantly, reinforcing its role not only in modulating stress responses but also in counteracting the effects of ABA and JA. This effect occurs through the *NPR1* gene, which strengthens SA signaling while suppressing the activation of ABA‐related genes (*NCED3* and *MYC2*) and JA‐related genes (*PDF1.2*) (La et al. 2019; Daszkowska‐Golec & Szarejko, 2013). In *C. wilsonii*, SA levels rose by approximately 4‐fold in diploids and 2‐fold in tetraploids compared to well‐watered controls, highlighting its involvement in drought tolerance through transcriptional and metabolic regulation (Jiang et al. 2022).

These hormones also influence photosynthetic activities and the biosynthesis of secondary metabolites, which are crucial for maintaining plant growth and productivity during drought stress (Ghassemi‐Golezani and Farhangi‐Abriz, 2021; Pirbalouti et al. 2019). JA and SA contribute to stress tolerance by altering the levels of phenylpropanoids and other metabolites. Additionally, SA affects root hydraulic properties by regulating aquaporins, which are essential for water transport in plants, helping them cope with reduced water availability during drought (Arbona et al. 2015; Argamasilla et al. 2013; Quiroga et al. 2017).

Given the pivotal role of root architecture, depth, and density in the citrus response to water deficit, particularly under low soil moisture conditions, there is growing interest in exploring drought resilience across various citrus scion/rootstock combinations (Romero et al. 2006; Carr, 2012; Santana‐Vieira et al. 2016; Shafqat et al. 2021). Rootstocks with deeper, more branched root systems demonstrate a greater capacity for water absorption in drier soils, as they can tap into water reserves inaccessible to plants with less developed root systems (Koevoets et al. 2016; Meneses et al. 2020; Sánchez‐Blanco et al. 2014). Studies such as Pedroso et al. (2014) have shown that the Cravo lime tree, a drought‐tolerant rootstock, relies on enhanced root growth and the remobilization of carbohydrates to the roots under water‐deficit conditions. This strategy enables greater water absorption and supports other physiological processes, underscoring the critical role of root and carbohydrate metabolism in drought tolerance.

Additionally, Meneses et al. (2020) highlighted the importance of fine and ephemeral roots in water uptake and hydraulic conductivity, further reinforcing the relevance of the root system in citrus drought resilience. Khalid et al. (2021) extended these findings by inferring lower root hydraulic conductivity in tetraploid citrus rootstocks based on indirect observations, including reduced transpiration and potassium translocation to leaves. These characteristics favor stomatal closure and water conservation, suggesting that drought tolerance in tetraploids may rely on mechanisms that balance water use efficiency with physiological adjustments. Together, these studies highlight the complex interaction between root system dynamics, hydraulic traits, and the physiological and biochemical processes of plants in shaping citrus drought tolerance strategies.

### 1.3 The Connection Between the Tetraploidization Process and Rootstock Drought Tolerance

Over the last decades, tetraploidization has emerged as a potentially significant factor in promoting drought resilience in tree crops, including apple, fig, citrus, and other species (Abdolinejad and Shekafandeh, 2022; Fonollá et al. 2023; Liao et al. 2024; Li et al. 2009; Wójcik et al. 2022). Comparative studies of different genotypes and tetraploid citrus rootstocks, though still limited, reinforced the complexity and variability of responses, indicating that drought tolerance in citrus is a multifactorial phenomenon involving an integrated network of different processes (Allario et al. 2013; Oliveira et al. 2017).

In a seminal study, Allario et al. (2013) reported that tetraploid Rangpur lime rootstocks exhibited greater drought tolerance than their diploid counterparts, a response partially explained by the higher root ABA production in tetraploid rootstocks. Gene expression analysis revealed that more genes related to water deficit response were differentially expressed in diploids than tetraploids, suggesting that plants with diploid rootstocks were more stressed than those with tetraploid rootstocks. Additionally, the increased expression of CsNCED1, which encodes the enzyme 9‐cis‐epoxycarotenoid dioxygenase involved in ABA biosynthesis, was detected in dry tetraploid roots compared to the diploid counterparts, which may explain the higher shoot ABA levels observed in these plants. The authors emphasize that the results support the interpretation that ABA signaling from the root to the shoot plays a crucial role in driving significant physiological changes in the shoot during drought stress. However, they highlight that ABA signaling alone may not be sufficient to fully explain these changes.

Subsequently, Oliveira et al. (2017) revealed a remarkable adaptive advantage of tetraploid'Carrizo citrange' rootstocks under drought conditions compared to their diploid equivalents. A combination of physiological and biochemical factors, including significantly lower water consumption, evidenced by reduced stomatal conductance, photosynthesis, and transpiration, was identified as responsible for allowing the tetraploid plants to withstand prolonged drought. Additionally, tetraploid rootstocks demonstrated an enhanced ability to neutralize ROS under water deficit, protecting cells and tissues from oxidative damage and ensuring plant functionality even under stress. Moreover, the expression of the antioxidant enzyme catalase 2 (CAT2)‐encoding gene was significantly higher in tetraploids, suggesting that activation of enzymatic antioxidant defenses conferred increased protection for tetraploid rootstocks by minimizing drought‐induced oxidative stress.

More recently, Wei et al. (2019) investigated the response of autotetraploid trifoliate rootstocks to water deficit, identifying increased drought and dehydration tolerance in autotetraploids compared to diploids. The tolerance was related to an enhanced ability to eliminate ROS and the accumulation of sugars, which act as osmoprotectants, helping to maintain cellular integrity. Transcriptomic analysis revealed that a gene encoding vacuolar invertase (VINV), responsible for the hydrolysis of sucrose into glucose and fructose, was significantly more expressed in tetraploids under drought stress. This increased VINV expression led to higher glucose accumulation in tetraploid rootstocks, possibly contributing to osmotic adjustment under drought conditions.

Comparative studies with'Kinnow' mandarin grafted onto diploid and tetraploid Volkamer lemon (Khalid et al. 2021), and sour orange diploids and tetraploids (Hussain et al. 2023), showed that in both cases, tetraploid plants maintained photosynthetic capacity, chlorophyll content, and cellular integrity under drought stress. This suggests a more robust photosynthetic performance and greater adaptability of tetraploids compared to diploids to water deficit. Moreover, tetraploids exhibited higher activity of antioxidant enzymes, such as superoxide dismutase (SOD) and CAT, and elevated levels of osmoprotectants, such as proline and betaine, compared to diploids. These compounds play a crucial role in cellular protection against osmotic stress and oxidative damage, contributing to the higher drought tolerance observed in tetraploid rootstocks.

In summary, studies by Allario et al. (2013), Oliveira et al. (2017), Wei et al. (2019), Khalid et al. (2021), and Hussain et al. (2023) suggest that tetraploid citrus genotypes and rootstocks exhibit greater drought tolerance compared to their diploid counterparts (Table 1). Complex and diversified responses were observed across these reports, as tetraploid citrus exhibited lower water consumption, enhanced antioxidant capacity, more efficient hormonal regulation, higher osmoprotectant accumulation and/or greater robustness of the photosynthetic system compared to diploids. Additionally, the increased stomatal regulation observed in tetraploid rootstock plants was associated with higher ABA levels, reinforcing the'Gigas' effect. This'Gigas' effect, characteristic of polyploidy, occurs due to the increased DNA content, leading to larger cells and impacting key physiological processes, such as the production of phytohormones, including ABA, which plays a crucial role in the plant response to water deficit (Becker et al. 2022; Sattle et al. 2016; Tan et al. 2017).

**TABLE 1:** Comparative Analysis of Physiological, Biochemical, and Growth Responses of Diploid and Tetraploid Citrus Plants Under Water Deficit Conditions: A Synthesis of Key Findings.

Feature	Diploid Plants	Plants Tetraploids	References
Water Consumption	Higher	Lower	Oliveira et al. (2017); da Silva Costa et al. 2024
Stomata Size	Smaller	Larger	Allario et al. (2013); Tan et al. (2015)
^1^ABA content in roots	Basal levels	Elevated levels (under water deficit)	Allario et al. (2013); da Silva Costa et al. 2024
Accumulation of compatible solutes	Lower	Higher	Wei et al. (2019); Hussain et al. (2023)
Antioxidant Enzyme Activity	Lower	Higher	Oliveira et al. (2017); Wei et al. (2019); Khalid et al. (2021); Hussain et al. (2023)
Chlorophyll Content	Significant reduction (under water deficit)	More stable (under water deficit)	Khalid et al. (2021); Hussain et al. (2023)
^2^ *VINV* gene expression	Basal levels	Higher expression (under water deficit)	Wei et al. (2019)
Photosynthesis Rate (under water stress)	Greater reduction	Lower reduction	Khalid et al. (2021); Hussain et al. (2023)
Root Growth	Greater	Lower	Allario et al. (2011); Guerra et al. (2014); Ruiz et al. (2016)
Graft size	Greater	Lower	Guerra et al. (2014); Jokari et al. (2022)
Drought Tolerance (greenhouse studies)	Variable	Variable (may be similar, superior or inferior to diploids, depending on conditions)	Oliveira et al. (2017); Allario et al. (2013); Wei et al. (2019); Khalid et al. (2021); Hussain et al. (2023); da Silva Costa et al. 2024
Drought Tolerance (field studies)	Greater	Lower	Espinoza‐Núñez et al. (2011); Oustric et al. (2020); Girardi et al. (2021)
Observations	‐	Responses may vary depending on rootstock ploidy, as the experimental conditions were the same for both.	

^1^ **ABA**: abscisic acid, a plant hormone associated with water stress response.

^2^ **VINV**: gene encoding the vacuolar invertase enzyme, related to sugar metabolism.

However, it is noteworthy that these experiments were conducted under different soil water potentials since diploid rootstocks display greater water extraction capacity compared to tetraploids (Allario et al. 2013; Oliveira et al. 2017; Wei et al. 2019), and attention to the interaction between tetraploidization and other environmental and genetic factors is urgently needed (Van Hieu, 2019). Studies performed in pots should consider soil water as a primary factor, as diploid and tetraploid citrus plants respond differently to water limitation. Differential water absorption in soil, influenced by specific root system characteristics and water use efficiency, may lead to evaluations at distinct matric potentials. As increasingly reported across drought resilience studies (da Silva Costa et al. 2024, Moshelion et al. 2024, Sousa et al. 2024), this can result in misleading conclusions about stress response strategies, especially when transpiration dynamics and soil water extraction capacity are not adequately considered. Additionally, variability in plant responses at different soil moisture levels highlights the importance of careful time‐course monitoring throughout the stress period to avoid erroneous interpretations that could compromise extrapolating results to field conditions.

For example, contrary to previous studies, da Silva Costa et al. (2024) demonstrated that'Sunki Tropical' tetraploid rootstocks did not show increased drought tolerance when grown in competition with diploids under the same soil moisture conditions. The primary evidence was the manifestation of water stress symptoms, such as leaf wilting, at a similar period as the diploid rootstocks. Additionally, tetraploids showed increased leaf ABA levels, indicating a more intense response to drought than diploids. Sucrose levels decreased in tetraploids under competition and drought, while sucrose, fructose, and glucose levels increased in the diploids. These responses suggest that, under competition, tetraploids struggled to maintain their drought tolerance strategy, possibly due to the greater water absorption capacity of diploids, which have more extensive and finer root systems.

Therefore, under competition with diploids for limited soil resources, citrus tetraploids lose their ability to handle drought, displaying more sensitive responses to water deficit than diploids (da Silva Costa et al. 2024). For instance, tetraploids exhibited higher oxidative damage when grown in separate pots, evidenced by elevated hydrogen peroxide accumulation compared to diploids. In future research comparing tetraploid and diploid rootstocks, it is crucial to consider differences in plant transpiration, root water extraction, and soil matric potential. This approach will ensure a more accurate assessment of the most likely drought responses of each genotype under field conditions, avoiding potential misinterpretations.

### 1.4 Real‐World Performance of Tetraploid Rootstocks: A Field Perspective

As discussed above, despite the predominant assumption that tetraploid citrus rootstocks can enhance drought tolerance compared to diploids in controlled conditions (Table 1), these conclusions are based on assessments conducted under different soil matric potentials and limited time‐course analysis throughout the stress imposition period. Although still poorly investigated, data indicates that tetraploid citrus, in actual field conditions, are more sensitive to drought than diploids. Oustric et al. (2020) and Girardi et al. (2021) explored the performance of tetraploid rootstocks in field conditions, contrasting with the results observed in greenhouse studies. Oustric et al. (2020) evaluated the drought tolerance of common clementine grafted onto different rootstocks by assessing physiological (Ψpd, RWC, gas exchange, and chlorophyll fluorescence), biochemical (oxidative markers and antioxidant enzyme activity), and agronomic parameters (fruit yield, size, and quality). Interestingly, the tetraploid rootstock (FlhorAG1) did not improve the drought tolerance of clementine compared to its diploid counterpart and other diploid genotypes, such as'Carrizo' citrange, under mild water deficit in field conditions. The mild water deficit was associated with a predawn leaf water potential (Ψpd) ranging from approximately ‐0.9 MPa to ‐1.6 MPa, with higher Ψpd values observed in seed‐propagated rootstocks and lower values in cuttings and the allotetraploid FlhorAG1. This observation contrasts with previously described higher tolerance to abiotic stress of tetraploids under greenhouse studies.

Moreover, Girardi et al. (2021) assessed the growth and production of'Valencia' sweet orange grafted onto various rootstocks, including a tetraploid selection of Swingle citrumelo, in dryland cultivation under an Aw climate (i.e., tropical savanna climate with dry winters). Although the tetraploid selection reduced tree size, its performance under drought conditions was unsatisfactory. The tetraploid Swingle citrumelo failed to sustain adequate productivity without supplemental irrigation, displaying low water use efficiency and reduced drought tolerance compared to the other rootstocks evaluated.

**FIGURE 3:**
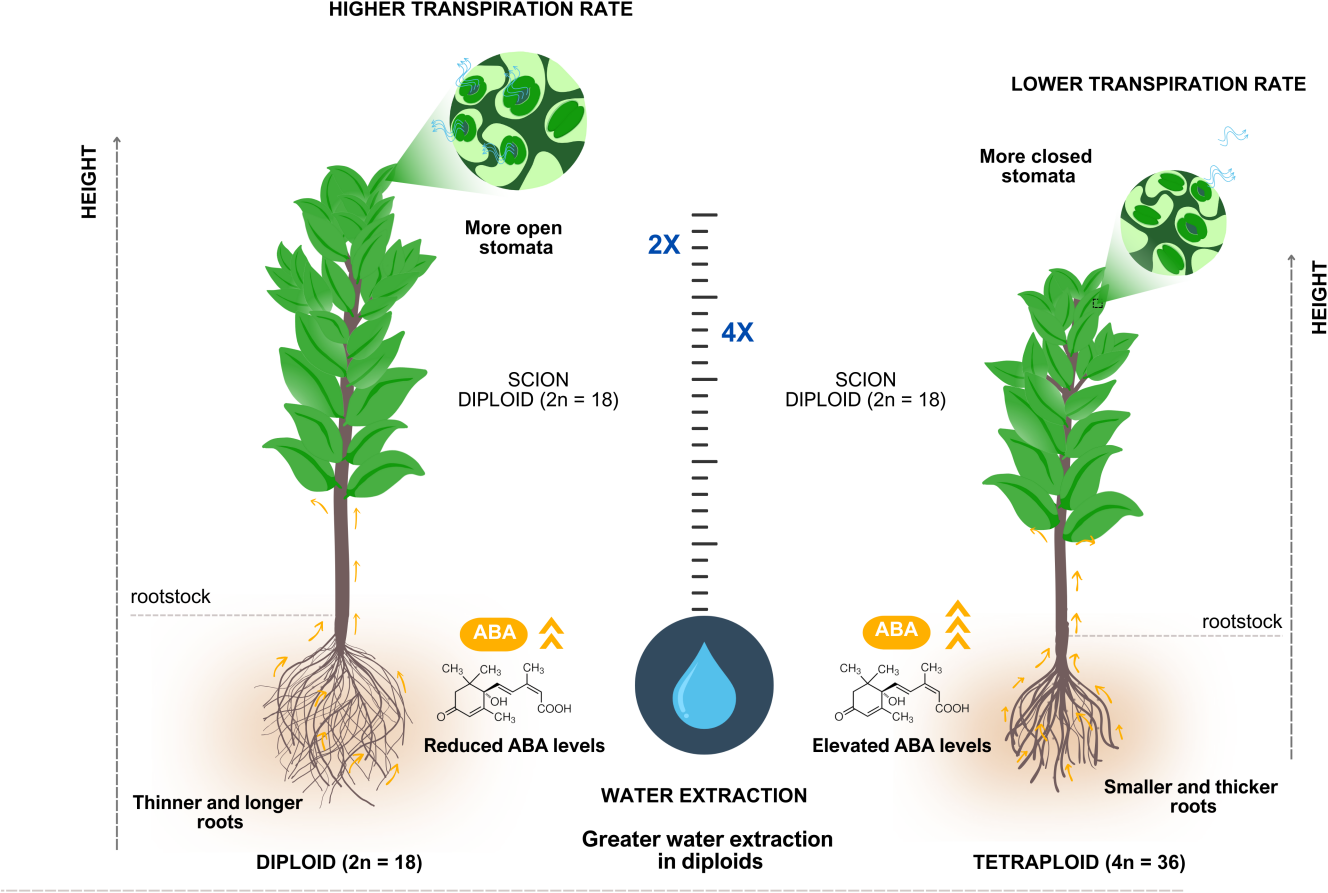
Comparison of citrus plants grafted onto diploid and tetraploid rootstocks – Citrus plants grafted onto diploid rootstocks (left) develop thinner and deeper roots, which enhance water extraction from the soil, and produce lower levels of abscisic acid (ABA). This results in more open stomata and a higher transpiration rate. In contrast, plants grafted onto tetraploid rootstocks (right) develop shorter and thicker roots, which extract less water from the soil. These plants produce higher levels of ABA, leading to greater stomatal closure and a lower transpiration rate compared to plants grafted onto diploid rootstocks.

The discrepancy between greenhouse and field results can be attributed to various factors. First, it is crucial to consider the morphophysiological differences between genotypes/rootstocks and ensure similar soil water potential (da Silva Costa et al. 2024; Sousa et al. 2024). Tetraploid rootstocks, with greater stomatal regulation and smaller, thicker roots, tend to be more water‐efficient than diploids (Figure 3). This difference is reflected in the rate at which each type absorbs water from the soil, as demonstrated by Allario et al. (2013), where diploids showed signs of water deficit earlier due to faster consumption of available water. However, drought tolerance does not necessarily translate into more conservative water use, as evidenced by Corso et al. (2015) and Opazo et al. (2020), who observed higher transpiration rates in more drought‐tolerant rootstocks due to efficient root hydraulic conductivity, delayed stomatal closure, and regulated aquaporin expression. Opazo et al. (2020), for instance, demonstrated that these traits allowed plants grafted onto ‘R40’ to sustain water transport, transpiration, and photosynthesis for longer periods, optimizing water use under water deficit conditions. These findings emphasize the complexity of drought tolerance and the need to consider physiological and morphological traits to understand water use efficiency under different conditions.

However, the difficulty in extrapolating data from controlled environments to field conditions is not limited to assessments of drought resilience. In controlled environments, conditions are rigorously monitored, which does not represent the complexity and variability of field conditions (Sprenger et al. 2016). Factors such as temperature, humidity, solar radiation, and soil interactions, which can vary significantly in the field, influence plant responses to water deficits, leading to divergences from results observed in controlled environments (Deikman et al. 2012; Mahalingam et al. 2015; Zandalinas and Mittler, 2022). Additionally, in the case of grafted plants, the interaction between the rootstock and the canopy may differ in field conditions. The canopy, with its water and nutrient demands, may influence the responses within the rootstock tissues to adjust water uptake and transport, and this dynamic may not be fully replicated in greenhouse studies (Valverdi and Kalcsits, 2021).

Another crucial aspect to consider is the duration of drought stress. Greenhouse studies often impose short‐term stress, while in field conditions, plants may face prolonged and more severe water deficits (Bhattacharya, 2021; Chaves et al. 2002). This difference in stress duration can significantly affect the response of tetraploid rootstocks, which may exhibit different tolerance mechanisms at different stages of stress. Additionally, citrus plants growing in pots are usually subjected to restricted root growth and water absorption compared to those under field conditions (Sinclair et al. 2017). Field plants often have access to deeper water reserves in the soil profile than potted plants. Exploring a larger soil volume for water allows field plants to withstand much more extended drought than potted plants, with limited available water (Turner, 2019; Vadez et al. 2011). Tetraploid rootstocks have morphological traits that allow for greater water economy, such as smaller roots and higher ABA production, which may be advantageous during moderate and short‐duration drought under pot conditions. Conversely, diploids tend to consume more water, and at a faster timeframe, due to their more extensive root systems and higher absorption capacity (Figure 3). However, under prolonged and severe droughts, the tolerance of tetraploids may be compromised, giving an advantage to diploids due to their greater water absorption capacity (Guerra et al. 2014; Ruiz et al. 2016).

Despite the lack of studies directly comparing the performance of tetraploid rootstocks with their diploid counterparts in pots versus in the field, the evidence discussed above indicates markedly different results obtained in the field and pot conditions, even with traditional rootstocks. A notable example is the study by Santos et al. (2019), which revealed a discrepancy between field and pot results regarding drought tolerance of'Cravo' lime and'Sunki Maravilha' tangerine. In the field,'Cravo' lime without grafting was significantly more drought tolerant than'Sunki Maravilha' tangerine without grafting, contrasting with observations in pot studies. Additionally, grafted plants, particularly self‐grafted'Cravo' lime, respond differently to water deficits, showing limitations in photosynthesis and changes in antioxidant metabolism. This not only highlights the need for caution when extrapolating pot study results to the field but also indicates that further research is required to deepen our understanding of the complex interactions between rootstocks, canopy, and environmental conditions, especially regarding water stress.

Guidelines for improving the quality of drought experiments in controlled conditions are emerging, emphasizing rigorous experimental design that accounts for genotype‐environment interactions, sufficient biological replications, and precise control of variables like soil water status and microclimate (Moshelion et al. 2024; Osmolovskaya et al. 2018; Ogbaga et al. 2020; Wang et al. 2024). Avoiding biases from factors such as plant transpiration is critical, as these can distort results and perpetuate misconceptions, like the belief that tetraploid rootstocks consistently outperform diploids in drought tolerance. Findings have shown no advantage for tetraploids when grown alongside diploids under the same soil moisture conditions (da Silva Costa et al. 2024), although such studies emphasize below‐ground competition for water that may not occur in production environments. These insights highlight the importance of considering factors like gas exchange, water consumption, and soil matric potential, even in separate pots, to ensure comparable soil moisture. Controlled studies, when carefully designed to simulate stress duration, intensity, and competition, can reliably reflect field‐like responses.

Although the performance of tetraploid rootstocks under field water restriction conditions is generally lower than that of diploid rootstocks, they still show great potential for sustainable applications, provided they are properly managed with efficient irrigation strategies, such as drip irrigation, soil moisture monitoring, and irrigation scheduling based on weather conditions to optimize water use and ensure adequate plant growth under water‐limited conditions. Furthermore, these rootstocks attenuate canopy vigour, resulting in plants of smaller size. This characteristic not only facilitates higher planting density but also reduces operational costs, such as those related to harvesting. In this context, Espinoza‐Núñez et al. (2011) demonstrated that, under non‐irrigated conditions, the'Carrizo' and'Troyer' tetraploid citranges had lower accumulated yield and productive efficiency than other rootstocks evaluated. On the other hand, when cultivated under irrigation, these same rootstocks showed significant increases in accumulated yield, with more than 3‐fold increases. Thus, even with limitations under water stress, tetraploids, when combined with careful water management practices, offer a sustainable solution that can promote greater agricultural resilience in the face of climate change while contributing to more rational water use in citrus cultivation.

## 2 CONCLUSIONS AND FUTURE PERSPECTIVES

In diploid citrus plants, drought tolerance is associated with complex responses involving stomatal closure, ABA production, gene expression reprogramming, changes in carbohydrate metabolism, and increased antioxidant defenses. In addition, root morphology also proves crucial, with deeper and more branched roots providing greater water absorption capacity in dry soils. Although the mechanisms of drought tolerance in diploid citrus rootstocks are widely studied and explored, the stress response of their tetraploid counterparts still requires in‐depth investigation. Studies in controlled environments suggest that tetraploid rootstocks exhibit greater drought tolerance than diploids; however, these conclusions are influenced by experimental limitations, leading to misinterpretations and overestimating the drought tolerance of tetraploids. To ensure the accuracy of results in comparative studies, it is essential to conduct experiments under controlled conditions, with the same soil matric potential in pots, avoiding assessments under different water potentials. Methodologies such as those proposed by Sousa et al. (2024) and da Silva Costa et al. (2024), along with the recommendations suggested by Moshelion et al. (2024), are recommended for a more rigorous and reliable evaluation of rootstocks under drought conditions, contributing to the development of more efficient and resilient agricultural practices. The influence of factors such as the duration and intensity of drought stress, competition for resources, and interactions with diseases and pests must be rigorously analyzed, as these elements can significantly alter the responses of rootstocks. Furthermore, a comprehensive understanding of the response of tetraploid rootstocks to drought and the development of effective strategies to enhance citrus resilience requires robust field studies and multidisciplinary approaches integrating plant physiology, genetics, and bioinformatics.

To enhance the sustainability of citrus cultivation in the face of climate change, future research on tetraploid rootstocks should prioritize improving traditional methodological aspects, such as monitoring plant water status and standardizing soil moisture. Additionally, it is crucial to intensify the search for specific genes and metabolic pathways that confer drought tolerance in these plants. In this context, the CRISPR/Cas9 gene‐editing technology offers remarkable potential for improving drought resilience in citrus rootstocks. By enabling precise genetic modifications, CRISPR can facilitate the development of plants with enhanced stress tolerance, optimized root architecture, and improved nutrient absorption traits essential for surviving water scarcity (Chauhan et al. 2025; Li et al. 2019; Wang et al. 2019). The precision, efficiency, and simplicity of CRISPR make it a superior tool compared to traditional breeding methods, accelerating the development of climate‐resilient cultivars (Shahid et al. 2024). Adopting these innovative approaches will not only allow for a more precise selection of adaptable rootstocks but also improve fruit productivity and quality characteristics, thereby strengthening the sustainability of citrus agriculture in the context of climate change.

## AUTHOR CONTRIBUTIONS

L.S.C, M.A.A.S, and F.S.N. collected information and prepared the manuscript. A.S.G, M.A.C.F, and L.F reviewed and organized the manuscript. All authors contributed to the article and approved the submitted version.

## FUNDING INFORMATION

This work was supported by Embrapa (20.22.01.005.00.00).

## CONFLICT OF INTEREST STATEMENT

The authors declare no conflict of interest.
